# A randomized comparative study on successional versus premixed intrathecal administration of hyperbaric ropivacaine 0.75% and fentanyl in elective urological surgery

**DOI:** 10.6026/973206300213649

**Published:** 2025-10-31

**Authors:** Tania Ghosh, Sapna Bansal, Hersimran Kaur, Ayman Deeba, Tarjani Ayachit, Tarjani Khiani, Vijul Chawla, Chandni, Rizwana Shaheen, Arathy Anilkumar

**Affiliations:** 1 Department of Anaesthesia, M.M. Institute of Medical Sciences & Research, Mullana, Ambala, Punjab, India

**Keywords:** Ropivacaine, fentanyl, successional, premixed, spinal anaesthesia, urological surgery

## Abstract

The effects of successional versus premixed administration of fentanyl and hyperbaric ropivacaine for urological surgeries is of interest. Hence, a total of 56 patients were randomized into two groups: Group P received a premixed solution and Group S received fentanyl followed by ropivacaine. Results showed that Group S had an earlier onset and longer duration of sensory and motor blocks, with more stable hemodynamics. Side effects were comparable between the two groups. Thus, we show that successional administration provides faster onset, prolonged block duration and better hemodynamic stability with minimal side effects.

## Background:

Spinal anaesthesia is increasingly utilized for lower abdominal and urological surgeries due to its cost-effectiveness, reduced complication rates and rapid recovery, making it a preferred option for many patients [1]. The most commonly used local anaesthetics for spinal anaesthesia include bupivacaine, lidocaine and tetracaine [2]. Typically, 0.5% hyperbaric bupivacaine is employed for its moderate onset (5-8 minutes) and long duration (1.5-2 hours). The introduction of hyperbaric ropivacaine (0.75%), an entirely natural S-enantiomer, has significantly improved safety by offering lower cardiotoxicity, faster motor recovery, reduced CNS toxicity and more precise sensorimotor differential blockade compared to bupivacaine [3]. Fentanyl, a lipophilic opioid, enhances post-operative pain relief (180-240 minutes) when administered intrathecally, though it may cause side effects like itching, nausea and respiratory depression [4]. The addition of fentanyl to hyperbaric bupivacaine alters the solution's baricity, which can influence the spread of local anaesthesia within the cerebrospinal fluid (CSF). While hyperbaric solutions offer stable hemodynamics and prolonged analgesia, a change in baricity by as little as 0.0006 can significantly affect the distribution of the anaesthetic [5, 6]. This study aimed to compare the effectiveness of successional versus premixed delivery of intrathecal hyperbaric ropivacaine 0.75% and fentanyl in patients undergoing elective urological surgeries. The comparison focused on the onset and duration of sensory and motor blocks, the maximum sensory block level achieved, the degree and duration of motor block and the hemodynamic changes observed in both groups after drug administration. Additionally, side effects were assessed in each group. Therefore, it is of interest to determine the optimal method of administration, contributing to improved anaesthetic practices in urological surgeries under spinal anaesthesia.

## Methodology:

This prospective and randomized study was conducted after taking clearance from Institutional Ethical Committee (project no: IEC-2575) and registering with the Clinical Trial Registry India-CTRI/2024/02/063018. A total of 56 patients of either gender of age 18-65 years and ASA I and ASA II status scheduled for elective urological surgery under spinal anaesthesia were included in study after obtaining consent for the study. Patients with coagulopathy, infection at injection site, hypotension, disease or deformity of spine, psychiatric illness and known sensitivity to the drug were excluded from the study. The patients were randomly divided into two groups of 28 patients each using a computer-generated programme into: Group P - premixed 0.75% hyperbaric ropivacaine 2.5ml (18.75 mg) and 0.5 ml (25 microgram) of fentanyl in a single 5.0 ml syringe. Group S - 0.5 ml (25 microgram) of fentanyl in a 2.0 ml syringe followed by 0.75% hyperbaric ropivacaine 2.5 ml (18.75 mg) in a 5.0 ml syringe. A detailed history, complete physical examination and routine & appropriate investigations was done for all patients. After a thorough pre-anaesthetic check-up, patients were shifted to operating room. The drug codes, sealed in envelops numbered 1-56, were opened by a designated fellow who was unaware of the study design and group allocation. Subarachnoid block was administered under all aseptic precautions using 25- gauge Quincke spinal needle at L3-4 interspace in sitting position according to the group by an anaesthesiologist with an experience of 5 years atleast. Patients were turned supine keeping the operation table horizontal. Onset of Sensory block was assessed by a sterile pin prick every 1 min till the highest level was achieved. Motor blockade of the lower limbs was tested and scored according to the modified Bromage scale.

Onset of sensory block was defined as the loss of sensation at T10 dermatome. The time of onset of motor block was defined as time taken to reach Modified Bromage score of 3. Similarly, time of regression of motor block was assessed from Modified Bromage score of 3 to score of 0. Variation in systolic blood pressure (SBP), diastolic blood pressure (DBP), mean arterial pressure (MAP), heart rate (HR), respiratory rate (RR), arterial oxygen saturation on pulse oximetry (SpO2) were assessed periodically till the completion of the procedure. Hypotension was defined as decrease of systolic blood pressure to 20% from baseline or more and was treated with 6 mg mephentermine intravenously. Bradycardia defined as HR <60 beats per minute was corrected using 0.6 mg of intravenous atropine sulphate. Side effects manifested by patients in intra and post operative period were also noted. Based on the sample size formula [n = (σ12 + σ22) (Zα + Zβ)2/ (m1 - m2)^2^] with 95% confidence and 99% power, 25 participants per group were required, increased to 28 per group (56 total) to account for dropouts. Data was presented as mean ± SD, requencies and percentages. Normality was tested with the Kolmogorov-Smirnov test. Student's t-test and Mann-Whitney test were used for parametric and non-parametric comparisons, respectively, while Chi-square and Fisher's exact test were used for categorical data. A p-value < 0.05 was considered significant and all calculations were performed using SPSS 21.

## Results:

The average age of patients (Table 1 - see PDF) in group S was 49.36±13 years, while in group P it was 47.82±12.3 years, with no significant difference in age between the two groups (p-value = 0.55). No notable statistical distinction turned out between the two groups in the distribution of age, gender distribution and comparison of ASA grading. The onset of sensory blockade was quicker and the duration of sensory block was longer in Group S compared to Group P. In Group S, complete motor blockade (Bromage 3) was attained earlier compared to Group P. Furthermore, Group S had substantially longer motor block duration than Group P, with a notable variation (p < 0.001) (Table 2 - see PDF). 25 (89.29%) patients in group P obtained the maximum level of sensory block - T6, compared to only 4 (14.29%) in group S. As a result, the groups differ statistically significantly in the highest level of sensory blockade. The fall in heart rate (Figure 1 - see PDF) was significantly more in group P compared to group S at 4 and 6 minutes post-spinal induction, with no significant differences at other time points up to 30 minutes. Group S had a statistically significantly higher mean arterial pressure (MAP) compared to Group P at 2 minutes (101.65±7.46 mmHg vs. 93.89±6.99 mmHg, p < 0.01), 4 minutes (99.74±5.55 mmHg vs. 93.31±8.15 mmHg, p = 0.001) and 6 minutes (99.13±6.12 mmHg vs. 89.94±7.04 mmHg, p = 0.001) (Figure 2 - see PDF). No significant changes were found in SpO2 levels and respiratory rate between the groups across all time intervals. It was noted that Group S did not exhibit any instances of bradycardia, whereas, bradycardia was observed among only 3 out of the 28 patients of Group P. Regarding itching/pruritus, 2 participants (7.14%) in Group S reported this symptom, while 3 participants (10.71%) in Group P. Notably, there were no occurrences of hypotension, vomiting, nausea, respiratory depression, or urine retention in either group.

## Discussion:

In most of the institutes, routine practice which is being followed in administration of local anaesthetic and adjuvant intrathecally for spinal anaesthesia is premixed i.e., local anaesthetic and adjuvant is mixed in a single syringe prior to administration. The most common local anaesthetic that is being used intrathecally is Hyperbaric (0.5 %) Bupivacaine and if adjuvant is added to it then it is in premixed fashion. This research was carried out to assess and incorporate successional method of drug administration to obtain more acceptable block, hemodynamic stability and reduced incidence of side effects. Previous researches have compared premixed and successive delivery of intrathecal fentanyl and bupivacaine. Recently, there has been a growing utilization of ropivacaine, an S-enantiomer of bupivacaine, for spinal anaesthesia in various surgeries, including cesarean sections, infraumbilical and perineal surgeries and lower extremity surgeries. Additionally, hyperbaric 0.75% ropivacaine is now commercially available in the market. Ropivacaine has several advantages over bupivacaine, including a shorter duration of motor block while keeping identical sensory blockade qualities. This shorter motor block duration helps in minimizing the psychological discomfort associated with prolonged immobility. Furthermore, ropivacaine exhibits lower cardiotoxicity compared to bupivacaine, which is another significant advantage [7]. Studies show that to achieve comparable anaesthesia effectiveness to 8mg of bupivacaine, administering 12mg of ropivacaine is required [8]. This signifies that equipotent dose of bupivacaine: ropivacaine is 1:1.5. Hence, in our study we took 2.5ml of 0.75% Hyperbaric Ropivacaine to conduct the study basing on research conducted with 0.5% hyperbaric bupivacaine. Successional group had a significantly earlier sensory block onset time than premixed group. Studies by El Kenany [9], Shukla *et al.* [10] found that sequential administration of fentanyl and hyperbaric bupivacaine resulted in a quicker onset of sensory block compared to mixing, supporting our findings on the efficacy of consecutive dosing. In our study, the premixed group attained a higher sensory block level in comparison to the successional group, a result parallel to the findings of Chekole *et al.* [11]. This outcome can be elucidated by noting that a mixed solution tends to generate an unpredictably higher level of block because when the solution is mixed, it disperses freely within the cerebrospinal fluid (CSF) without relying on gravity for dissemination as there is reduction of density of LA when mixed with opioid making the mixture hypobaric. In line with our findings, Malhotra *et al.*'s [12] study showed that administering fentanyl separately leads to a wider spread and stronger associations with opioid receptors in the spinal cord, resulting in a dense and extended duration of sensory block. All patients in our study achieved a Bromage 3 motor block but time to achieve was faster in sequential group. Routray *et al.* [13] observed similar results in their trial, where all patients receiving premixed ropivacaine with fentanyl achieved complete motor block (Bromage 3) within 10 minutes. However, Koppal *et al.* [14] reported that 80% of participants in the ropivacaine group reached Bromage 3. No research could be found with sequential administration of ropivacaine (0.75%) and fentanyl. In conclusion, our study found that the sequential administration group had earlier onset and longer duration of sensory and motor blocks compared to the premixed group. These effects are likely due to the mechanisms of intrathecal drug distribution, where the hyperbaric solution spreads along the lumbar spine's curvature when the patient shifts from sitting to supine. Premixing fentanyl with ropivacaine alters the baricity of the solution, affecting its efficacy and contributing to the observed outcomes.

No significant differences were observed in HR before induction, immediately after induction, or at most subsequent time points. However, at 4 and 6 minutes post-induction, notable differences emerged, indicating fall in HR more in premixed group as highest height of sensory blockade was achieved in premixed group (T6) compared to successional group.

Fall in mean arterial pressure (MAP) was statistically significant in premixed group between 2 and 6-minutes post-induction, with no notable differences at other times. This result suggests that the administration method affects immediate response to heart rate and MAP, but overall trends were similar between the groups due to ropivacaine's cardio-stable properties. Studies by Malhotra *et al.* [12], Chekole *et al.* [11], also support sequential administration for better hemodynamic stability. There were no significant differences in respiratory rate or oxygen saturation (SpO2) between the two groups. No one experienced Hypotension (a SBP decrease of 20% from baseline) and consequently, no interventions with 6mg mephentermine intravenously were required. These findings are consistent with the known drug profile of Ropivacaine, which is recognized for its lesser cardiotoxic effects. 3 patients in the premixed group experienced Bradycardia and no such occurrence happened in successional group patients. Shashikala *et al.* [15] accomplished a study comparing the outcomes of adding fentanyl and dexmedetomidine to hyperbaric ropivacaine for patients enduring infraumbilical surgeries and they discovered that bradycardia occurred in only one of the 30 patients who received premixed ropivacaine and fentanyl. None of the participants in our study experienced nausea, vomiting, respiratory depression, or urine retention as adverse effects. This may be attributed to the drug profile of ropivacaine, which allows for earlier patient mobilization due to its shorter duration of motor block compared to other local anaesthetics like bupivacaine. We were unable to measure the in vivo densities of the drugs after intrathecal injection. Additionally, temperature and injection speed, factors affecting drug distribution and effectiveness, could not be documented. ASA grade III and IV patients, who could have benefited more from hyperbaric ropivacaine due to its lower cardiotoxicity, were excluded from the study. Future researches can assess the effectiveness of sequentially administering fentanyl and hyperbaric ropivacaine at various rates. A study can also be conducted using other non - opioid adjuvants like dexmedetomidine or clonidine with hyperbaric Ropivacaine 0.75% or different doses of fentanyl can also be evaluated further.

## Conclusion:

To conclude successional administration of hyperbaric ropivacaine 0.75% and fentanyl can be preferred over premixed administration of the same for performing spinal anaesthesia as it leads to earlier onset and longer duration of sensory and motor block, hemodynamic stability with minimal side effects.
